# Supplementary material for: Spatial transcriptomic profiling uncovers the molecular effects of the neurotoxicant polychlorinated biphenyls (PCBs) in the brains of adult mice
Source: Mol Psychiatry. 2026 Jan 31;31(6):3257–70. doi: 10.1038/s41380-026-03466-x (PMC13190273; doi:10.1038/s41380-026-03466-x)
Supplement: Supplementary file 1 — Supplemental information [file 41380_2026_3466_MOESM1_ESM.docx]

**Supplementary information for**

**Spatial transcriptomic profiling uncovers the molecular effects of the neurotoxicant polychlorinated biphenyls (PCBs) in the brains of adult mice**

Budhaditya Basu^1,2^, Nicole M. Breese^3,4^, Sal Lombardi^1,2^, Hui Wang^3^, Xueshu Li^3^, Destiny Tiburcio^5^, Zachary Niemasz^1,2^, Stacy E. Beyer^1,2^, Laura E. Dean^3^, Rachel F. Marek^6^, Michal Toborek^5^, Hans-Joachim Lehmler^2,3,4,6*^, and Snehajyoti Chatterjee^1,2*^

^1^Department of Neuroscience and Pharmacology, Carver College of Medicine, University of Iowa, Iowa City, IA, USA

^2^Iowa Neuroscience Institute, University of Iowa, Iowa City, IA, USA

^3^Department of Occupational and Environmental Health, College of Public Health, University of Iowa, Iowa City, IA, USA

^4^Interdisciplinary Graduate Program in Human Toxicology, University of Iowa, Iowa City, IA, USA

^5^Department of Biochemistry and Molecular Biology, University of Miami Miller School of Medicine, Miami, FL, USA

^6^IIHR-Hydroscience and Engineering, University of Iowa, Iowa City, IA, USA

*Corresponding authors:

hans-joachim-lehmler@uiowa.edu and snehajyoti-chatterjee@uiowa.edu

**Supplementary Information**

Supplemental Figures, Supplemental Figure legends S1-10, and Supplemental Table legends S1-S10.

**Supplemental Figure S1.** Comparison of PCB profiles (**A**) detected in postmortem human brain tissue with (**B**) the theoretical HR-PCB mixture developed based on mixing technical Aroclor PCB mixtures and individual PCB congeners, and (**C**) the synthetic HR-PCB mixture prepared to match the theoretic PCB profile. The human brain PCB profile represents the average PCB profile in the cerebellum from 30 male and 42 female donors, aged 8 to 59 years (average age, 33 years). The PCB profile shown in panels (A) and (C) were determined by GC-MS/MS. Human tissue was obtained from the NIH Neurobiobank at the University of Maryland, Baltimore, MD. See the details of the data in dataset (Li, X. et al. 2025 https://doi.org/10.25820/data.007553).


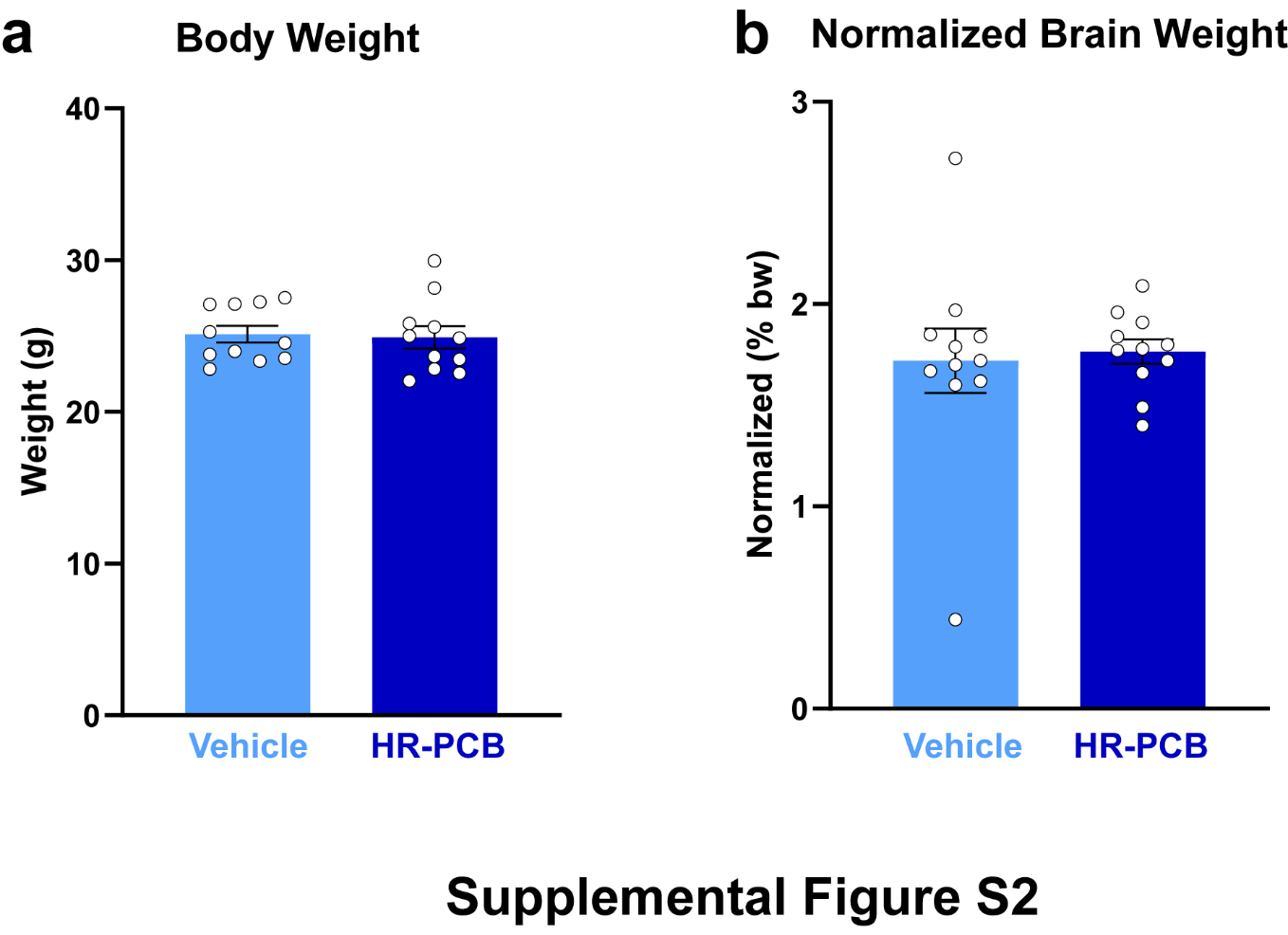


**Supplemental Figure S2. Body and brain weight assessment in vehicle and HR-PCB exposed animals.** Mouse body weight taken on the last day of PCB exposure. Net weight of brains adjusted for body weight of the mouse. Error bars represent ± SEM. Vehicle (n = 11), and HR-PCB (n = 11).

**
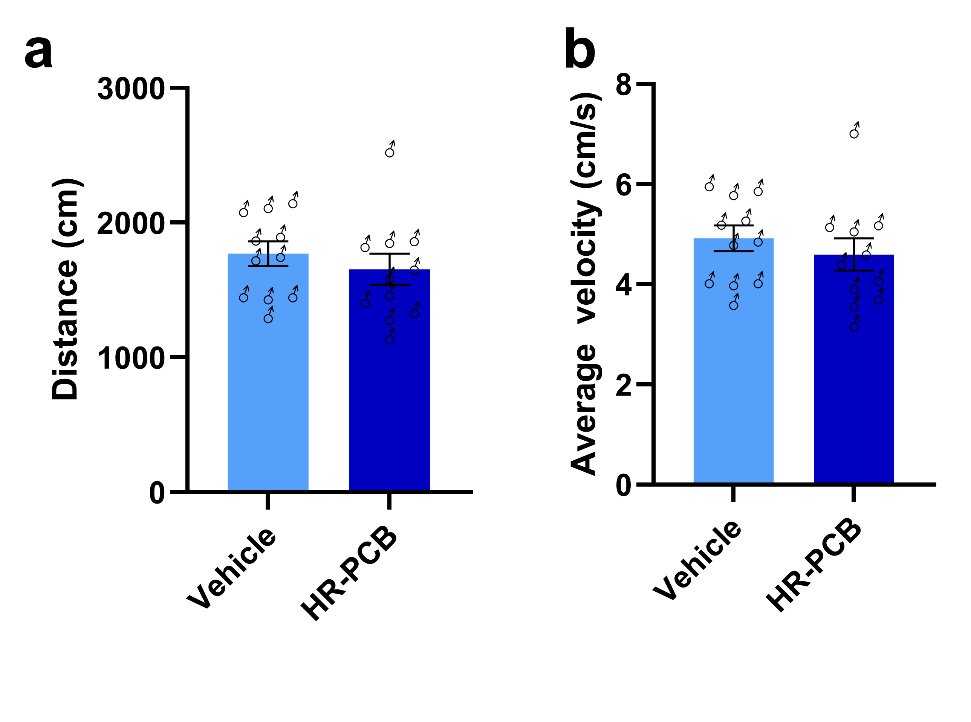
**

**Supplemental Figure S3:** Assessment of distance traveled and average velocity in vehicle and HR-PCB exposed animals during the habituation trial. Error bars represent ± SEM.

**
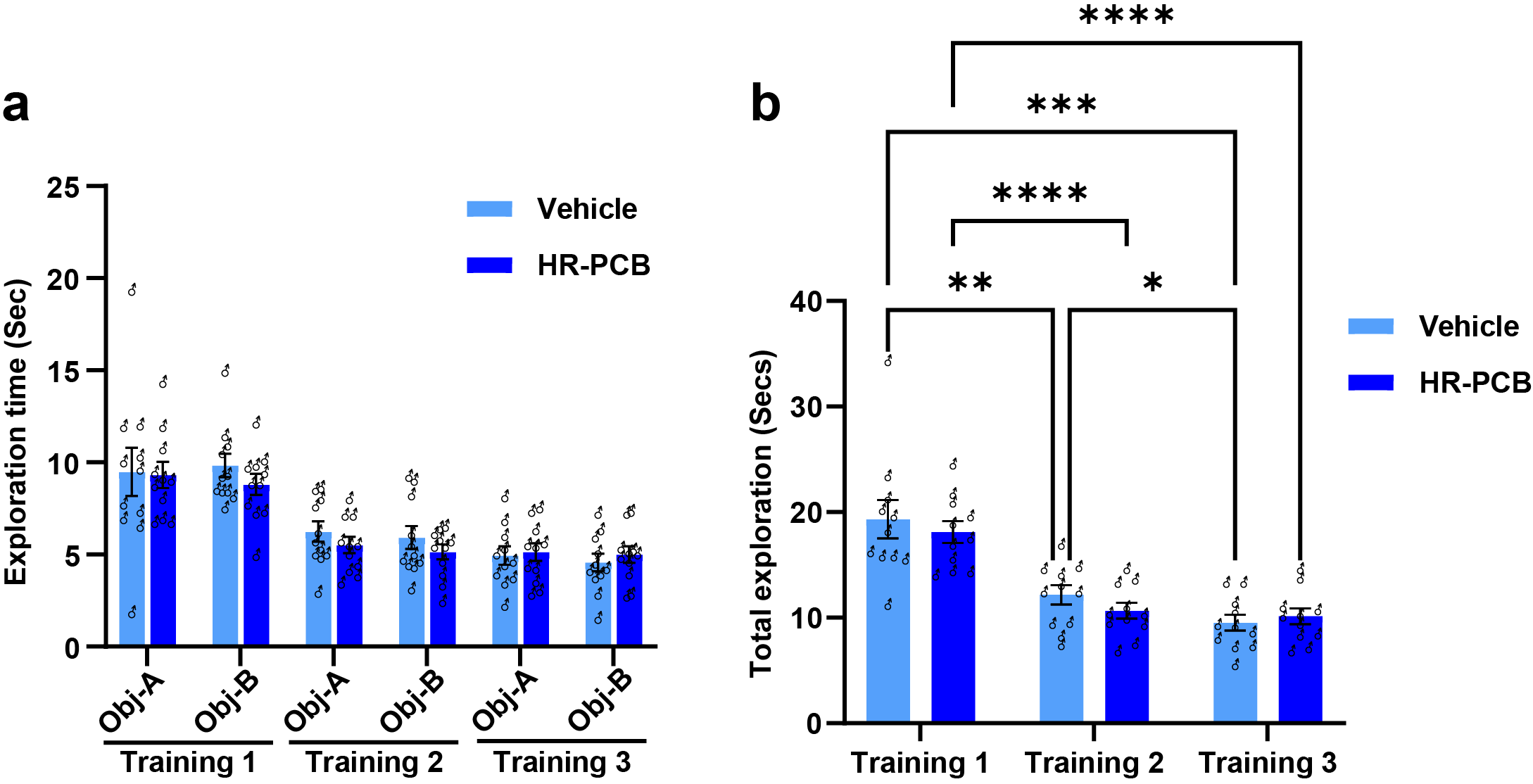
**

**Supplemental Figure S4**: (a) Bar graph showing the average time spent explorating the glass objects in the arenas across three training trials. (b) Bar graph depicting the total exploration time across three training trials. 2-way ANOVA: No significant interaction between training trials (Training 1, 2, 3) and exposure (vehicle-PCB): F (1.629, 32.58) = 0.8934, P = 0.3999; main effect of training trials: F (1.629, 32.58) = 61.45, P < 0.0001. Šídák’s multiple comparison tests: vehicle (Training 1) vs. vehicle (Training 2): **P = 0.0052; vehicle (Training 1) vs. vehicle (Training 3): ***P = 0.0004; vehicle (Training 2) vs. vehicle (Training 3): *P = 0.0417; HR-PCB (Training 1) vs. HR-PCB (Training 2): ****P < 0.0001; HR-PCB (Training 1) vs. HR-PCB (Training 3): ****P < 0.0001. Error bars represent ± SEM. Vehicle (n = 11), and HR-PCB (n = 11).

**
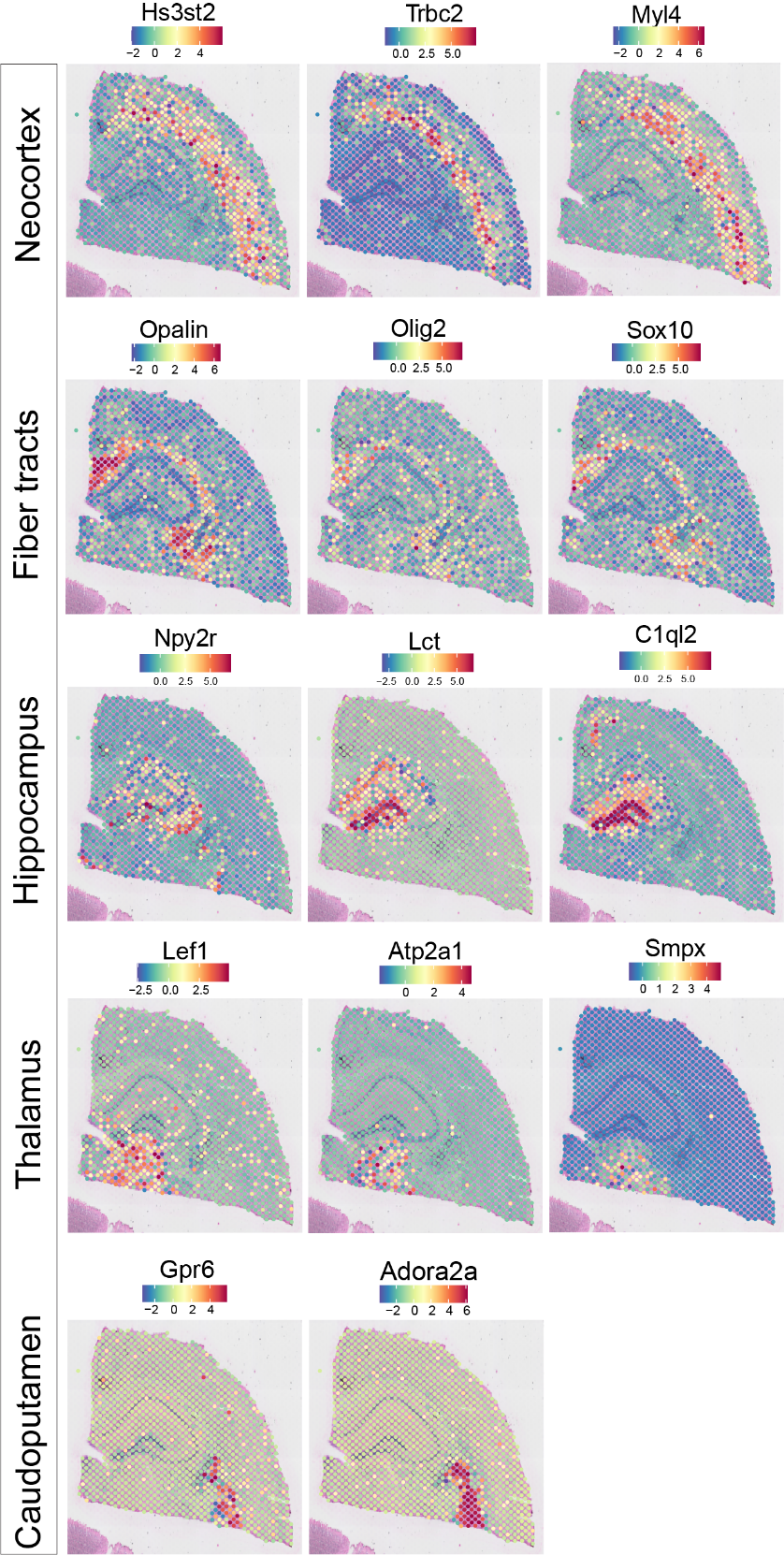
**

**Supplemental Figure S5:** Spatial mapping of canonical marker gene expression of five brain regions aligned on the H&E-stained image from the same tissue section.

**
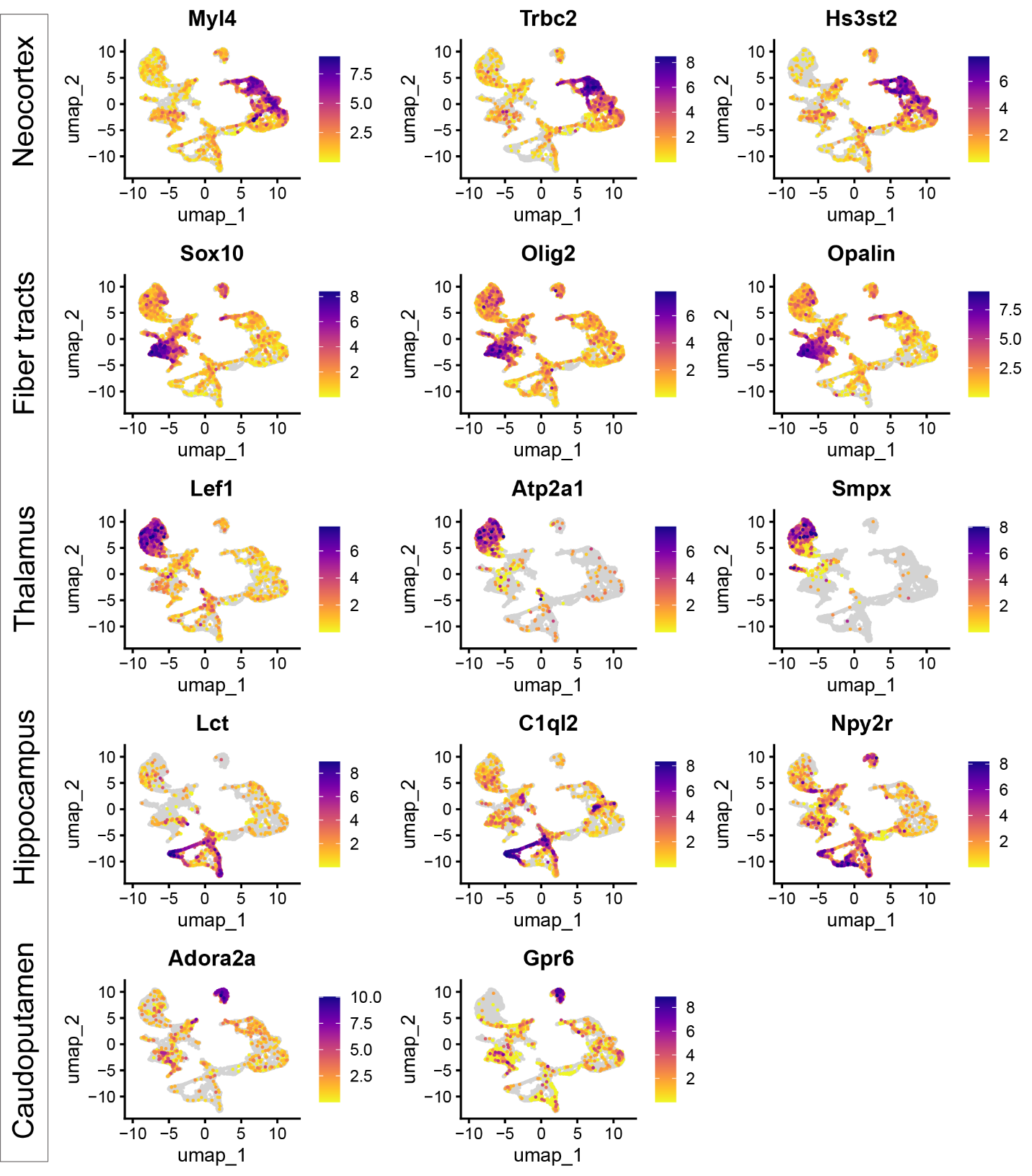
**

**Supplemental Figure S6:** Expression of the canonical marker genes of the five brain regions on UMAP projections.

**
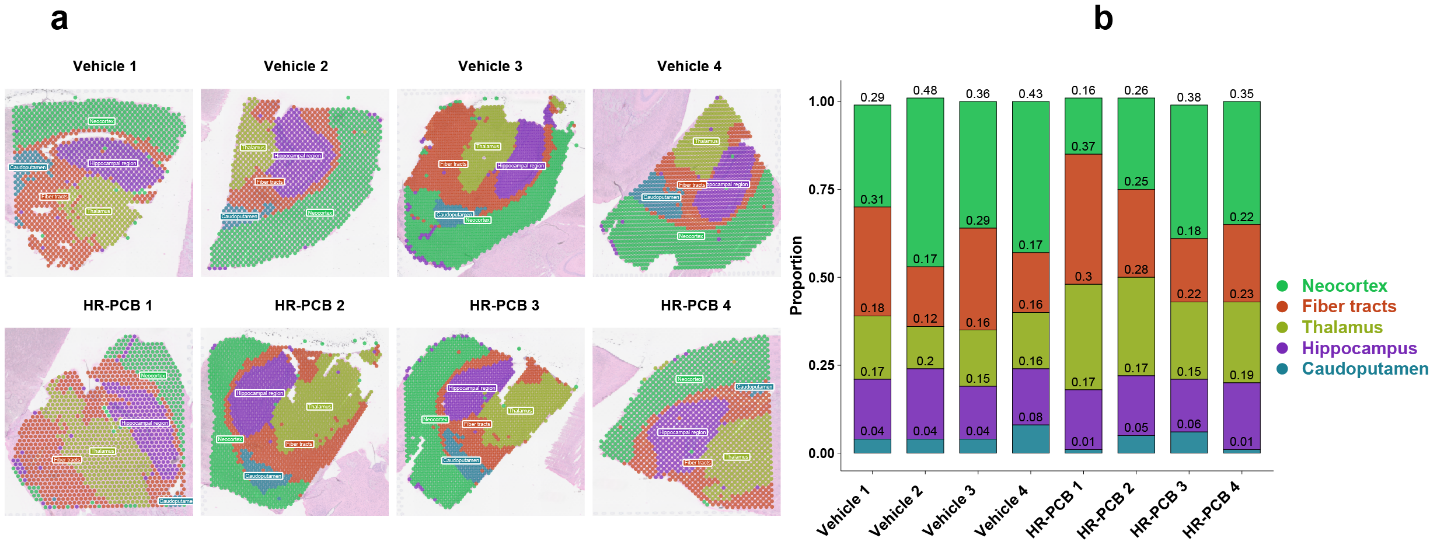
**

**Supplemental Figure S7. Brain section replicates for the Visium experiment. a.** Barcode spots (colored according to brain regions) overlayed on H&E-stained brain sections are shown for each biological replicate within each group. **b.** Bar plots showing the proportion of Visium spots across different brain regions between HR-PCB and vehicle-exposed animals. Mann-Whitney test comparing vehicle vs HR-PCB: Neocortex P-adj = 0.5 (ns), Fiber tracts P-adj = 0.857 (ns), Thalamus P-adj = 0.14 (ns), Hippocampus P-adj = 0.885 (ns), and Caudoputamen P-adj = 0.857(ns). Vehicle (n = 4 mice), and HR-PCB (n = 4 mice). ns: not significant.


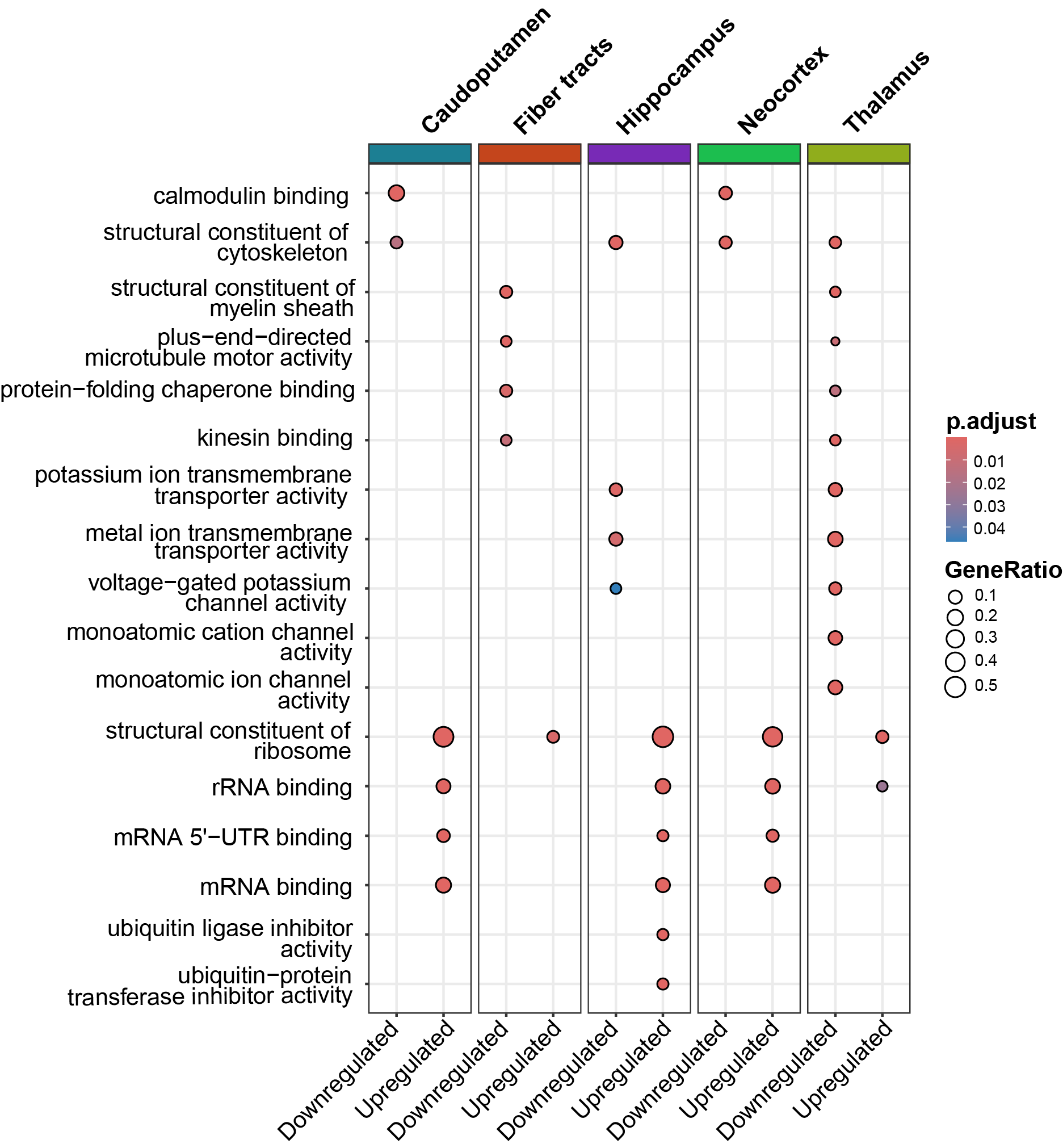


**Supplemental Figure S8:** Gene Ontology (Molecular Function) enrichment analysis by compareCluster function of ClusterProfiler.

**
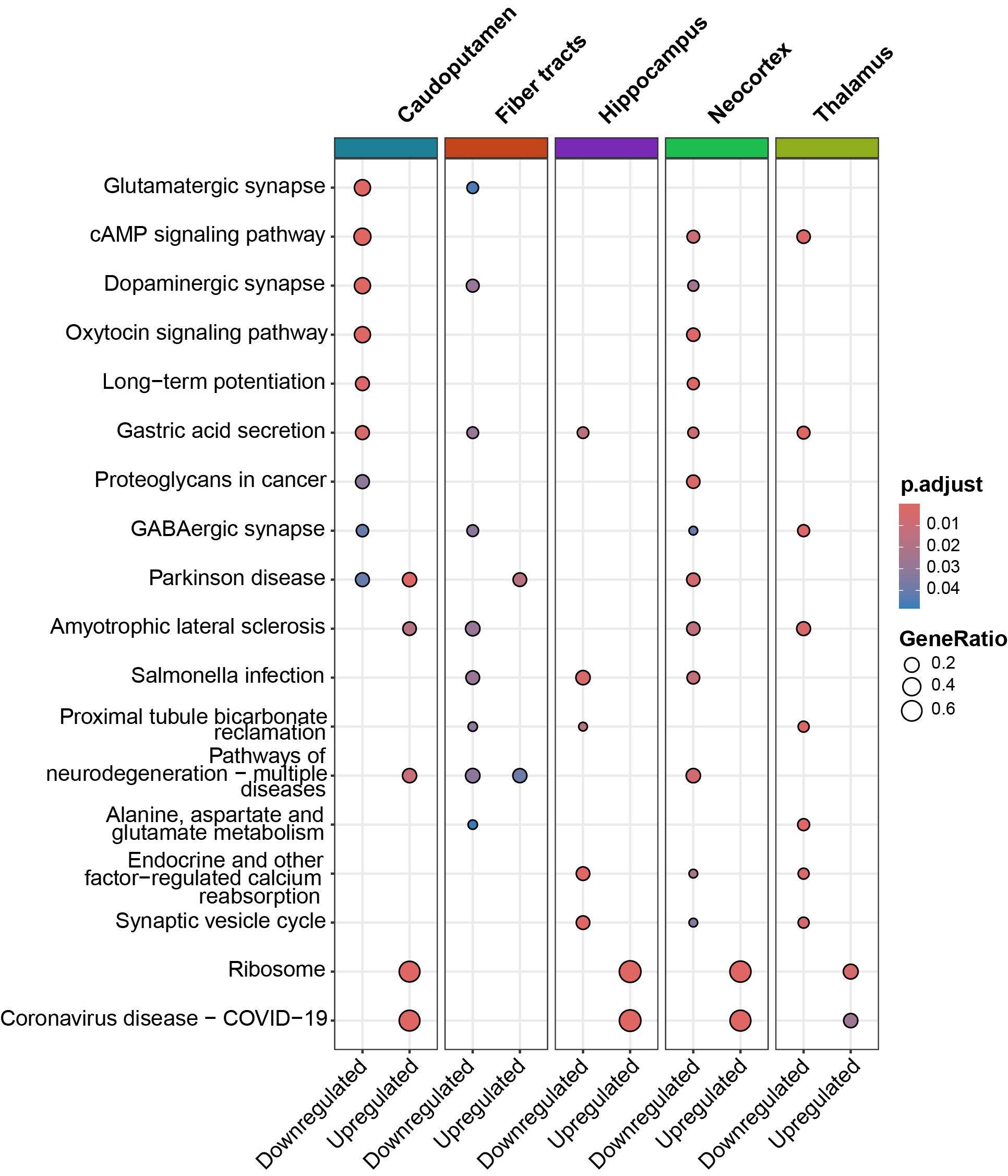
**

**Supplemental Figure S9:** KEGG pathway enrichment analysis by compareCluster function of ClusterProfiler.

**
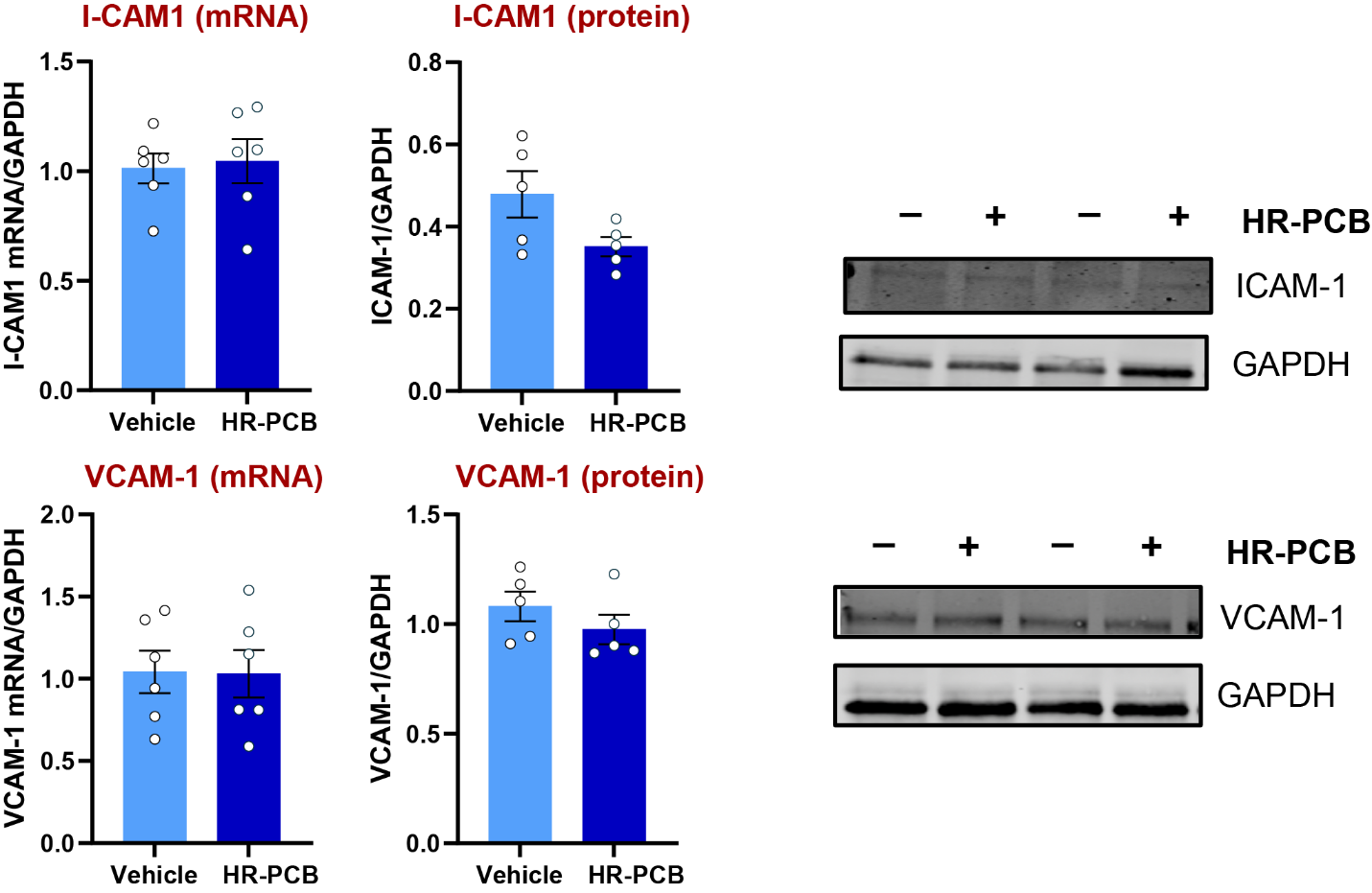
**

**Supplemental Figure S10. Expression of adhesion molecules ICAM-1 and VCAM-1.** Analyses were performed in whole brain homogenates of mice exposed to the HR-PCB mixture or vehicle control as in Figure 1. Left panels, RT-qPCR data. Middle and right panels, immunoblotting results presented as quantitative bar graphs and representative immunoblots of the target proteins. GAPDH levels were used to normalize the results. Values are mean ± SEM with n = 5-6 per group.

**Supplementary Table legends**

**Supplemental Table S1.** Surrogate standards recoveries for ^13^C labeled PCBs for each homolog group.

**Supplemental Table S2.** PCB precursor and product masses of unlabeled and ^13^C-labeled calibration standards employed in multiple reaction monitoring mode on the triple quadrupole mass spectrometer. Unlabeled standards were from AccuStandard, New Haven, CT, USA. Labeled standards were from Cambridge Isotope Laboratories, Inc.

**Supplemental Table S3.** Method Detection Limit (MDL) for each PCB congener or co-eluted congeners. MDLs were calculated using method blanks and expressed as the upper limit of 99% confidence interval (average + t_n-1_* standard deviation, t_n-1_ represents Student’s t-value of 99% confidence level with n-1 degree of freedom). Values are expressed in ng.

**Supplemental Table S4.** Limit of Detection (LOD) for each PCB congener or co-eluted congeners. LODs were calculated using control/blank tissues and expressed as the upper limit of 99% confidence interval (average + t_n-1_* standard deviation, t_n-1_ represents Student’s t-value of 99% confidence level with n-1 degree of freedom). Values are expressed in ng/g tissue.

**Supplemental Table S5**. PCB levels in the laboratory reference material (LRM). N = 7. RSD = SD/mean*100.

**Supplemental Table S6.** The difference of proportion across five brain regions was assessed between vehicle and HR-PCB group using Mann-Whitney test. A Benjamini & Hochberg correction method was applied to calculate the adjusted P-value.

**Supplemental Table S7.** Differentially expressed genes in five brain regions (neocortex, hippocampus, thalamus, fiber tracts, and caudoputamen).

**Supplemental Table S8.** Gene Ontology (Molecular Function) enrichment analysis on the DEGs across brain regions (Neocortex, hippocampus and thalamus).

**Supplemental Table S9.** Comparison of Gene Ontology (Molecular Function) and KEGG pathway enrichment on upregulated and downregulated genes across five brain regions.

**Supplemental Table S10.** Detailed quality metrics of Visium spatial gene expression.
